# miR-363 promotes proliferation and chemo-resistance of human gastric cancer via targeting of FBW7 ubiquitin ligase expression

**DOI:** 10.18632/oncotarget.9169

**Published:** 2016-05-04

**Authors:** Peng-Fei Zhang, Lu-Lu Sheng, Ge Wang, Mi Tian, Ling-Yin Zhu, Rui Zhang, Jing Zhang, Jin-Shui Zhu

**Affiliations:** ^1^ Department of Gastroenterology, Shanghai Jiao Tong University Affiliated Sixth People's Hospital, Shanghai 200233, P.R. China; ^2^ Department of Emergency Medicine, Shanghai Jiao Tong University Affiliated Sixth People's Hospital, Shanghai 200233, P.R. China

**Keywords:** gastric cancer, microRNA-363, proliferation, chemo-resistance, FBW7

## Abstract

Dysregulation of microRNA expression is involved in several pathological activities associated with gastric cancer progression and chemo-resistance. However, the role and molecular mechanisms of miR-363 in the progression and chemo-resistance of gastric cancer remain enigmatic. In this study, we validated that miR-363 expression was higher in gastric cancer tissues than in adjacent normal tissues. Multivariate analysis identifies high levels of miR-363 expression as an independent predictor for postoperative recurrence and lower overall survival. Increased miR-363 expression promotes gastric cancer cell proliferation and chemo-resistance through directly targeting the tumor suppressor F-box and WD repeat domain-containing 7 (FBW7). Clinically, our data reveal that overexpression of miR-363 correlates with the poor survival outcomes in patients with gastric cancer, and docetaxel + cisplatin + 5-FU (DCF) regimen response is impaired in patients with miR-363 overexpression. These data suggest that miR-363 may be a potential therapeutic target for gastric cancer and serve as a biomarker for predicting response to DCF regimen treatment.

## INTRODUCTION

Gastric cancer is the fourth most common cancer and second leading cause of cancer-related deaths worldwide [1]. Patients with advanced gastric cancer have a poor prognosis and the 5-year overall survival rate remains low at approximately 25% [1–3]. These dismal outcomes result from its high recurrence following curative stomach resection and its notorious resistance to systemic chemotherapy [4, 5]. In the past several decades, docetaxel + cisplatin + 5-FU (DCF) regimen has been used as a first-line treatment for advanced gastric cancer patients [6]. However, for patients with advanced gastric cancer, the response rate to neoadjuvant chemotherapy is more than 50% and nearly all patients develop chemotherapy resistance [7]. Thus, it is urgently needed to identify and develop new treatment strategies regarding gastric cancer molecular mechanisms involved in chemotherapy resistance.

microRNAs (miRNAs) belong to a class of short (19-22 nucleotides), noncoding RNA sequences [8]. It has been confirmed that aberrantly expressed miRNAs play essential roles in tumorigenesis and progression processes [9]. Recently, it has been reported that miR-940, miR-363, miR-25, and miR-269-5p function as oncogenes in gastric cancer [10–12], whereas miR-22, and miR-361-5p function as tumor suppress genes [13–15].

F-box and WD repeat domain-containing 7 (FBW7) is a substrate recognition component of the Skp1-Cul1-F-box (SCF) ubiquitin ligase complex and functions as a tumor suppressor by ubiquitination and degradation several oncoprotein proteins, including c-Myc, cyclin E, Notch-1, c-Jun, and Mcl-1, thus participating in inhibiting the progression of cancer [16–18]. Low expression of FBW7 is significantly correlated with poor prognosis in various human malignancies, such as gastric cancer, breast cancer, colon cancer, and esophageal squamous cell carcinoma [19–22]. However, the upstream signaling pathways that govern FBW7 stability and/or function are very limited [23].

In this study, we demonstrated that miR-363 expression is frequently increased in gastric cancer tissues and contributes to poor outcomes in clinical patient. Furthermore, the miR-363/FBW7 axis promotes the proliferation and chemo-resistance of gastric cancer cells. Moreover, these findings indicate that the miR-363/FBW7 axis is closely associated with the malignant phenotypes and serves as a potential therapeutic target for predicting response to DCF treatment in gastric cancer.

## RESULTS

### miR-363 is overexpressed in gastric cancer tissues, and a high level of miR-363 positively correlates with poor prognosis in gastric cancer patients

**Figure 1:**
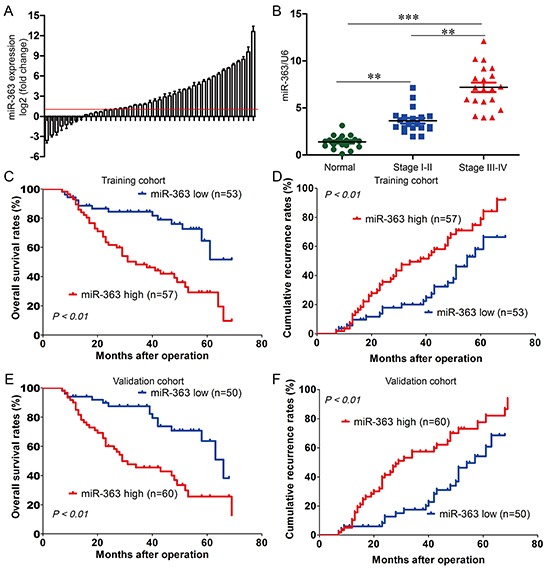
miR-363 is frequently increased in gastric cancer and significantly correlated with poor prognosis **A.** Expression of miR-363 in 110 pairs of gastric cancer tissues and their corresponding adjacent normal tissues in a training cohort. Expression level of miR-363 was determined by real-time PCR and normalized to U6. Red line indicates fold change of miR-363 equal to 2. **B.** 110 patients were divided into stage I-II and stage III-IV groups. The diagram shows the miR-363 expression of each group. **P<0.01, ***P<0.001. **C, D, E, and F.** Patients in the training and validation cohorts were divided into a miR-363^low^ expression group and a miR-363^high^ expression group, respectively, according to the result of real-time PCR. Survival curve was calculated with the log-rank test. Results show OS and recurrence of patients with high or low miR-363 expression in the training and validation cohorts.

It has been confirmed that miR-363 plays an important role in gastric carcinogenesis [11]. However, the relationship between gastric cancer and miR-363 expression remains elusive. To further explore the role of miR-363 in gastric cancer, we performed real-time PCR in 110 pairs of gastric cancer tissues and adjacent normal tissues in the training cohort. Compared with the corresponding adjacent normal tissues, miR-363 expression was significantly increased in 71 gastric cancer cases (64.5%; Figure 1A). These data suggested that increased miR-363 expression might be a frequent event in gastric cancers. In clinical stages, miR-363 expression in cancer tissues of stages III and IV was significantly higher than in cancer tissues of stages I and II (Figure 1B) (*P < 0.01*).

To examine the relationship between miR-363 expression and clinicopathological features, we analyzed their correlations in the training cohort. The results showed that miR-363 expression was significantly associated with poor clinicopathological features, including histologic grade (*P = 0.018*) (Supplementary Table S1). For further analysis, patients were separated into groups with high (n = 57) and low (n = 53) miR-363 expression groups. There was a striking, and statistically significant inverse association between miR-363 expression intensity and overall survival (OS; *P < 0.01*) (Figure 1C), and to a less extent, a statistically positive association between miR-363 expression intensity and cumulative recurrence (*P < 0.01*) (Figure 1D). Multivariate analysis revealed that miR-363 expression in tumors was an independent predictor for both OS and cumulative recurrence (Supplementary Table S2). Therefore, miR-363 expression is a valuable predictor for recurrence and survival in gastric cancer patients. These associations were further validated in another cohort comprised of 110 postoperative gastric cancer patients. Similarly, shorter OS and higher cumulative recurrence in the high miR-363 expression group than those in the low expression group were also observed (Figure 1E and 1F) (Supplementary Table S3). These results revealed that elevated expression of miR-363 was correlated with poor prognosis of gastric cancer, implicating that miRNA-363 was involved in gastric cancer progression.

### miR-363 promotes proliferation and chemo-resistance of gastric cancer cells *in vitro*

**Figure 2:**
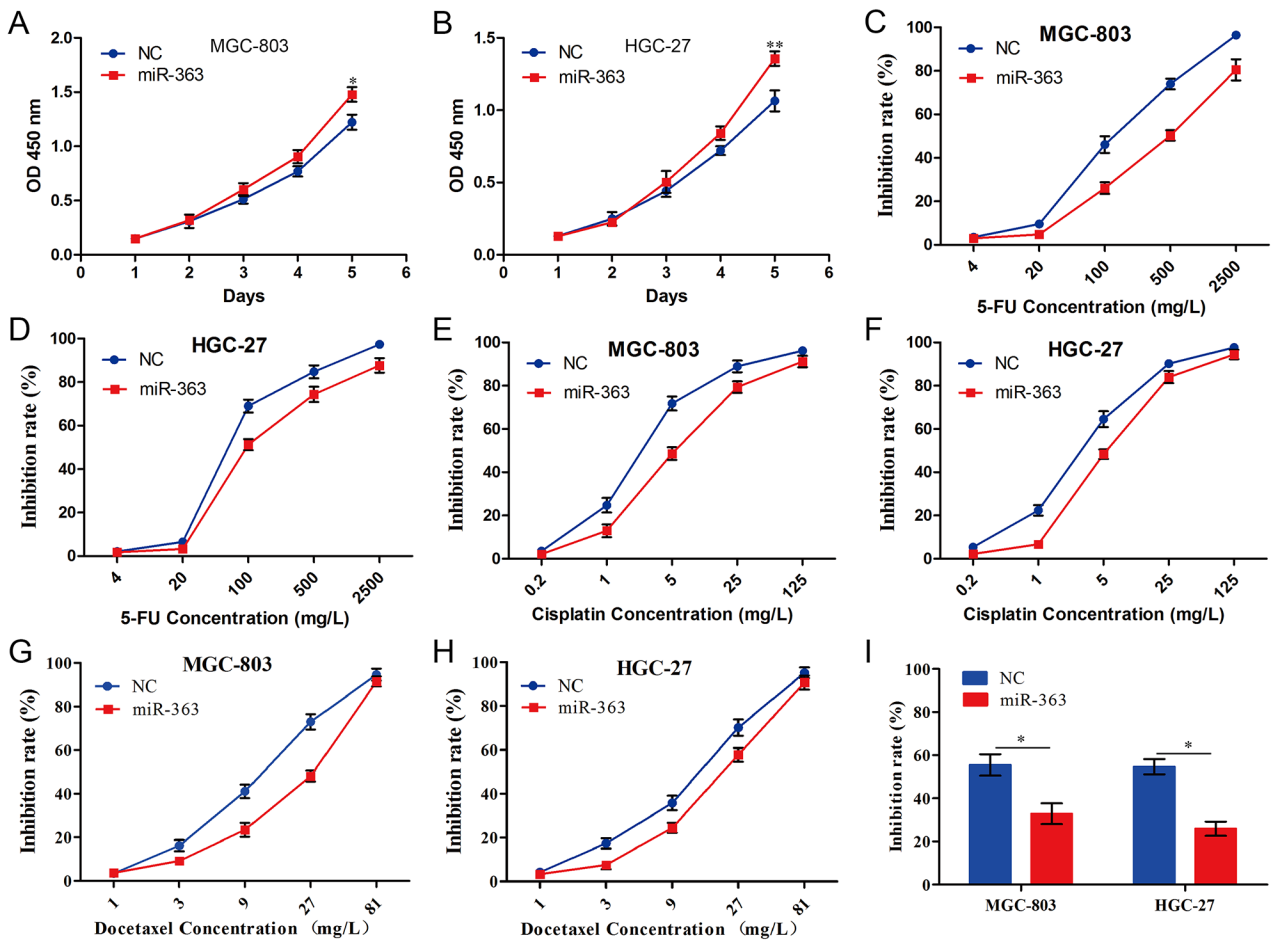
miR-363 promotes proliferation and chemotherapy resistance of gastric cancer cells *in vitro* Gastric cancer cell lines infected with miR-363 expression lentivirus or NC were used in these studies. **A and B.** Cell growth of gastric cancer cells was examined using CCK-8 assays. **C-H.** Forced miR-363 expression in gastric cancer cells reduced their sensitivity to 5-FU, cisplatin, and docetaxel. **I.** Forced miR-363 expression in gastric cancer cells reduced their sensitivity to low concentration DCF regimen (5-FU 20 mg/L, cisplatin 1 mg/L, and docetaxel 3 mg/L). Data are represented as the mean ± SD, n=3. *P<0.05; **P<0.01.

To elucidate the roles of miR-363 in gastric cancer cells, we generated MGC-803 and HGC-27 cells (which expressed lower levels of miR-363) overexpressing miR-363 (Supplementary Figure S1). Forced miR-363 expression significantly increased the proliferation abilities of MGC-803 and HGC-27 cells compared with the NC group (Figure 2A and 2B). Furthermore, we examined the role of miR-363 in gastric cancer cells sensitive to chemotherapy agents. For MGC-803-NC and MGC-803-miR-363 cells, the 5-FU IC_50_ values were 128.00 ± 16.31 mg/L and 463.83 ± 63.1 mg/L, respectively (*P<0.05*) (Figure 2C); for HGC-27-NC and HGC-27-miR-363 cells, the 5-FU IC_50_ values were 104.2 ± 2.68 mg/L and 231.55 ± 12.41 mg/L, respectively (*P<0.05*) (Figure 2D). For MGC-803-NC and MGC-803-miR-363 cells, the cisplatin IC_50_ values were 3.46 ± 0.23 mg/L and 7.83 ± 1.23 mg/L, respectively (*P<0.05*) (Figure 2E); for HGC-27-NC and HGC-27-miR-363 cells, the cisplatin IC_50_ values were 3.09 ± 0.41 mg/L and 7.19 ± 0.82 mg/L, respectively (*P<0.05*) (Figure 2F). For MGC-803-NC and MGC-803-miR-363 cells, the docetaxel IC_50_ values were 11.06 ± 0.80 mg/L and 18.53 ± 1.73 mg/L, respectively (*P<0.05*) (Figure 2G); for HGC-27-NC and HGC-27-miR-363 cells, the docetaxel IC_50_ values were 11.17 ± 1.52 mg/L and 17.92 ± 1.73 mg/L, respectively (*P<0.05*) (Figure 2H). Next, we examined the effects of low concentration DCF regimen (5-FU 20 mg/L, cisplatin 1 mg/L, and docetaxel 3 mg/L) on cell viability of MGC-803-NC, MGC-803-miR-363, HGC-27-NC, and HGC-27-miR-363 cells. Our data illustrated that a high level of miR-363 expression led to DCF regimen resistance in gastric cancer cells (*P<0.05*) (Figure 2I).

### FBW7 is a novel target of miR-363

**Figure 3:**
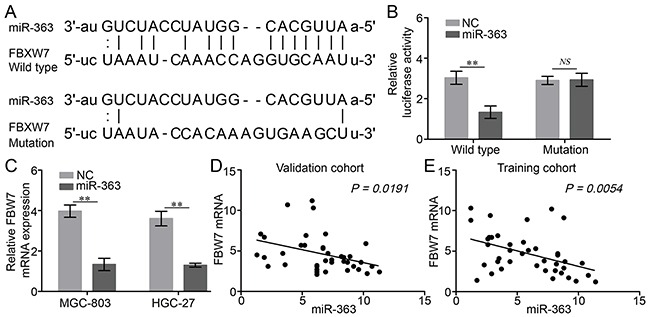
miR-363 directly targets FBW7 expression in gastric cancer cells **A.** Predicted target sequences in 3′-UTR of FBW7 bound to miR-363. **B.** The wild type or mutated FBW7 3′-UTR was transfected into gastric cancer cells with or without synthetic miR-363 mimic. Luciferase activity was determined 48 h after transfection. Data were normalized to the luciferase activity transfected with miRNA negative control (n=3). **C.** Expression of FBW7 mRNA upon miR-363 overexpression was detected in two gastric cancer cell lines by real-time PCR. **D and E.** A negative correlation between miR-363 and FBW7 mRNA was observed in gastric cancer tissues. Data are represented as the mean ± SD, n=3. **P<0.01.

To identify the potential target mRNA of miRNA-363, the databases TargetScan, PicTar, miRDB, and microcosm were used. We found that the 3′-UTR of FBW7 mRNA contains a sequence motif, which perfectly matching with the “seed-sequence” of the miR-363 (Figure 3A).

FBW7 is the substrate recognition component of the SCF ubiquitin ligase complex and functions as a tumor suppressor by targeting various oncoproteins for ubiquitination and degradation [16]. Therefore, we hypothesized that miRNA-363 might be capable of inhibiting the expression of FBW7, thereby promoting the proliferation and resistance to chemotherapy agents. To confirm whether FBW7 is a direct target of miR-363 in gastric cancer cells, we conducted luciferase reporter assays after transfection of wild type or mutated FBW7 3′-UTR into gastric cancer MGC-803 cells, with synthetic miR-363 mimic. We found that miR-363 reduced luciferase activity in cells transfected with wt FBW7 3′-UTR, but had no effect on luciferase activity in cells transfected with mutant FBW7 3′-UTR (Figure 3B). Additionally, forced expression of miR-363 reduced FBW7 mRNA levels in gastric cancer MGC-803 and HGC-27 cells (Figure 3C). Furthermore, the expression of miR-363 in human gastric cancer tissues was negatively related to FBW7 mRNA level (Figure 3D and 3E). Collectively, these results indicate that FBW7 is a direct target of miR-363 in gastric cancer.

### Knockdown of FBW7 expression promotes proliferation and chemo-resistance of gastric cancer cells *in vitro*

**Figure 4:**
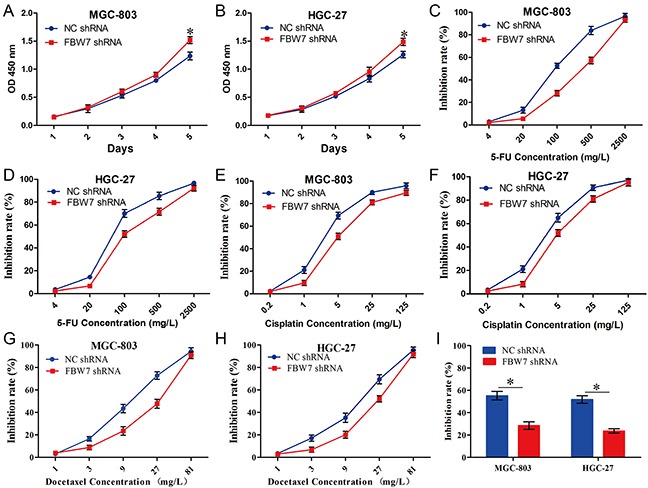
FBW7-shRNA promoted proliferation and chemo-resistance of gastric cancer cells *in vitro* Gastric cancer cell lines infected with FBW7-shRNA expression lentivirus or NC-shRNA were used in these studies. **A and B.** Cell growth of gastric cancer cells was examined with CCK-8 assays. **C-H.** Knockdown of FBW7 expression in gastric cancer cells reduced their sensitivity to 5-FU, cisplatin, and docetaxel. **I.** Knockdown of FBW7 expression in gastric cancer cells reduced their sensitivity to low concentration DCF regimen (5-FU 20 mg/L, cisplatin 1 mg/L, and docetaxel 3 mg/L). Data are represented as the mean ± SD, n=3. *P<0.05; **P<0.01.

To further confirm the hypothesis that miR-363 overexpression leads to comparative gastric cancer proliferation and resistance to chemotherapy agents by targeting FBW7, we generated FBW7 knockdown MGC-803 and HGC-27 cells. Knockdown of FBW7 expression significantly reduced the proliferation abilities of MGC-803 and HGC-27 cells compared with the NC-shRNA group (Figure 4A and 4B). Furthermore, we examined the role of FBW7-shRNA in gastric cancer cells sensitive to chemotherapy agents. For MGC-803-NC-shRNA and MGC-803-FBW7-shRNA cells, the 5-FU IC_50_ values were 104.27 ± 12.56 mg/L and 252.79 ± 42.12 mg/L, respectively (*P<0.05*) (Figure 4C); for HGC-27-NC-shRNA and HGC-27-FBW7-shRNA cells, the 5-FU IC_50_ values were 83.36 ± 7.06 mg/L and 180.14 ± 22.65 mg/L, respectively (*P<0.05*) (Figure 4D). For MGC-803- NC-shRNA and MGC-803-FBW7-shRNA cells, the cisplatin IC_50_ values were 3.98 ± 0.37 mg/L and 8.3 ± 0.37 mg/L, respectively (*P<0.05*) (Figure 4E); for HGC-27-NC-shRNA and HGC-27-FBW7-shRNA cells, the cisplatin IC_50_ values were 3.58 ± 0.44 mg/L and 6.6 ± 0.82 mg/L, respectively (*P<0.05*) (Figure 4F). For MGC-803- NC-shRNA and MGC-803-FBW7-shRNA cells, the docetaxel IC_50_ values were 11.06 ± 0.67 mg/L and 19.07 ± 1.73 mg/L, respectively (*P<0.05*) (Figure 4G); for HGC-27-NC-shRNA and HGC-27-FBW7-shRNA cells, the docetaxel IC_50_ values were 11.34 ± 1.57 mg/L and 18.46 ± 2.05 mg/L, respectively (*P<0.05*) (Figure 4H). Next, we examined the effects of low concentration DCF regimen (5-FU 20 mg/L, cisplatin 1 mg/L, and docetaxel 3 mg/L) on cell viability of MGC-803-NC-shRNA, MGC-803-FBW7-shRNA, HGC-27-NC-shRNA, and HGC-27-FBW7-shRNA cells. Our data illustrated that a low level of FBW7 expression led to DCF regimen resistance in gastric cancer cells (*P<0.05*) (Figure 4I). Together, these data suggest that miR-363 overexpression promotes gastric cancer cell resistance to chemotherapy agents by targeting FBW7.

### miR-363 exerts its function by suppressing FBW7 expression

**Figure 5:**
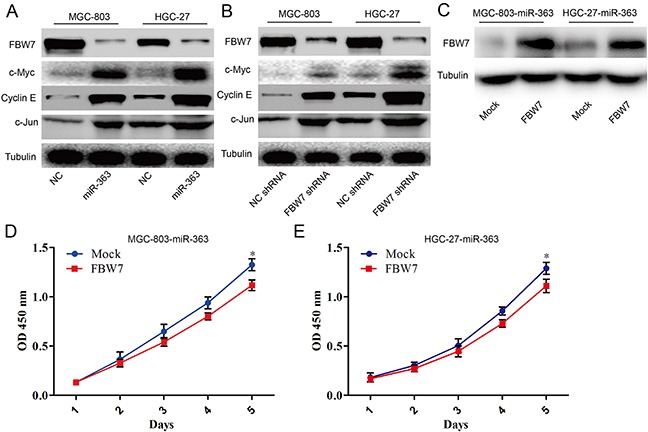
miR-363 overexpression inhibits FBW7 signaling in gastric cancer cells *in vitro* **A.** Forced miR-363 expression reduced FBW7 expression while enhanced c-Myc, c-Jun, and cyclin E expression in gastric cancer cells. **B.** Knockdown of FBW7 expression increased protein expression of c-Myc, c-Jun, and cyclin E in gastric cancer cells. **C.** Overexpressing FBW7 in MGC-803-miR-363 and HGC-27-miR-363 cells using a lentiviral vector. **D and E.** Cell growth of gastric cancer cells was examined with CCK-8 assays.

It has been confirmed that critical oncogenic FBW7 substrates including c-Myc, Mcl-1, cyclin E, and c-Jun. In order to better understand the mechanisms of miR-363 involved in proliferation and resistance to chemotherapy agents in gastric cancer cells, western blotting was used to detect the FBW7-shRNA- and miR-363-induced signaling pathway changes in MGC-803 and HGC-27 cells. Interestingly, as well as miR-363, FBW7-shRNA significantly inhibits c-Myc, Notch, cyclin E, and c-Jun expression at the protein level in MGC-803 and HGC-27 cells (Figure 5A and 5B). Next, we investigated the proliferation of the MGC-803-miR-363 and HGC-27-miR-363 cells with forced FBW7 expression and found that increased FBW7 expression was sufficient to block the miR-363-induced proliferation in gastric cancer cells (Figure 5C-5E).

### miR-363 expression is inversely correlated with gastric cancer sensitivity to DCF

**Figure 6:**
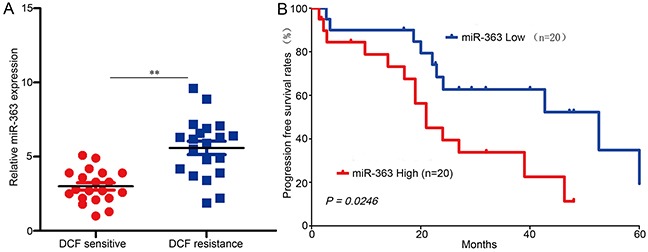
miR-363 induces DCF resistance in gastric cancer patients **A.** 40 patients were divided into DCF-sensitive and DCF-resistant groups. The diagram shows the miR-363 expression of each group. **P<0.01. **B.** Comparison of PFS survival curves for patients with high and low miR-363 expression that were treated with DCF.

We then analyzed retrospective data from 40 advanced recurrent gastric cancer patients receiving DCF therapy who had undergone gastric cancer resection 2 to 60 months prior to the combined therapy; patient demographics (Supplementary Table S4) and progression free survival (PFS) rates were recorded. miR-363 expression levels were then measured (Figure 6A), and Kaplan-Meier survival analysis indicated that the OS probability for the miR-363-high group was much lower than that for the miR-363-low group (Figure 6B). The median OS was 21.0 months in the miR-363-high group and 52.6 months in the miR-363-low group; therefore, we conclude that high levels of miR-363 expression lead to gastric cancer DCF resistance.

## DISCUSSION

Currently, DCF regimen remains a first line treatment course for patients with advanced gastric cancer. Chemo-resistance causing treatment failure and recurrence is still an intractable problem. As the poor prognosis in gastric cancer patients is mainly due to late diagnosis and bad chemotherapy response, thus, it is urgently needed to identify predictive markers of therapeutic response.

Recently, miRNAs have emerged as important biomolecules in regulating the responses of cancer cells to therapy agents. Patient response to chemotherapy has been proved closely correlated with the dysregualton of microRNAs expression [24–26]. Currently evidences suggests the roles of miRNAs, including influence of therapeutic induced cell apoptosis, regulation of multiple drug resistance (MDR)-related proteins expression, and induction of cancer cells converting to tumor stem-like cells (TSC) in several cancers [27]. Recently, studies have demonstrated that there is an intimate correlation between dysregulation of FBW7 expression and the incurring chemotherapy resistance. FBW7 exerts its tumor suppressor function by promoting the ubiquitination and degradation of various oncoproteins, including c-Myc, Mcl-1, cyclin E, and c-Jun, which regulate many cellular processes, for example, cell proliferation, cellular metabolism, differentiation, and apoptosis. Since dysregulation of the FBW7 expression is linked to chemotherapy resistance, FBW7 may represent a novel therapeutic target for increasing chemotherapy sensitivity of cancer cells to chemotherapeutics [28]. In this study, we showed that miR-363 promotes gastric cancer cells proliferation by inhibiting FBW7 expression and was associated with chemo-resistance of gastric cancer cells. Silencing FBW7 largely phenocopied miR-363-induced resistance to chemotherapy agents and promoted proliferation in gastric cancer cells. In addition, an inverse correlation between miR-363 and FBW7 mRNA expression was observed in gastric cancer tissues.

In summary, our findings indicate that increased miR-363 expression is a frequent event in gastric cancers and increased miR-363 expression promotes cell proliferation, chemo-resistance by inhibiting the FBW7 pathway, suggesting that targeting miR-363 in a subset of gastric cancer would be an optimal therapeutic strategy and miR-363 may be a biomarker for predicting responsiveness to chemotherapy treatment.

## MATERIALS AND METHODS

### Cell lines and clinical samples

Human gastric cancer HGC-27, MGC-803, SGC-7901, and BGC-823 cell lines were purchased from the Cell Bank, Shanghai Institutes for Biological Sciences, Chinese Academy of Sciences, Shanghai. NCI-N87 and AGS cell lines were purchased from the American Type Culture Collection. All of the cell lines were routinely maintained. Human gastric cancer tissues and adjacent normal tissues were obtained from the Shanghai Jiao Tong University Affiliated Sixth People's Hospital (Clinicopathological characteristics were listed in Supplementary Table S5). Sample collections were performed after receiving approval from the institutional ethics review committee of the Shanghai Jiao Tong University Affiliated Sixth People's Hospital, and no patients had undergone chemotherapy prior to surgery. Surgical evaluation was used to determine the clinical stage and pathologists performed histopathologic analysis to assess the cancer type and grade.

### RNA extraction, real-time PCR, and western blotting

Total RNA extraction, real-time PCR, and western blotting were performed as previously described [29, 30]. The sequence of primers for PCR used is listed in Supplementary Table S2.

### Cell proliferation assays

Cell proliferation was determined using a CCK-8 (Dojindo Laboratories, Kumamoto, Japan) performed as previously described [30].

### Drug sensitivity assay

For drug sensitivity assay, cells were seeded in 96-well plates at densities of 2 × 10^3^ cells per well. After 24 h, cells were placed in complete medium containing the indicated concentrations of docetaxel, cisplatin or 5-FU for 72 h, the sensitivity of the cells to docetaxel, cisplatin, 5-FU or DCF regimen was measured using CCK-8 Kit.

### Lentivirus production and transduction of target cells

microRNAs up-regulation was achieved by using a lentivector-mediated pseudoviral system (LV500A-1; System Biosciences, San Francisco, CA) performed as previously described [31]. The FBW7 and FBW7-shRNA expression lentivirus were purchased from Shanghai GeneChem Co., and the lentiviral vector was transfected into cells as described elsewhere [32].

### Luciferase reporter assays

Luciferase reporter assays were conducted using the Dual-Luciferase Reporter Assay System performed as previously described [30].

### Statistical analysis

Statistical analysis was performed with SPSS software (18.0; SPSS, Inc., Chicago, IL). The Student't test, Chi-square, Fisher's exact tests, Correlation analysis, Kaplan-Meier's method, and the log-rank test were used as described in the Supplementary Materials and Methods Values are expressed as mean±standard deviation (SD). P<0.05 was considered statistically significant.

## SUPPLEMENTARY MATERIALS FIGURES AND TABLES













## Abbreviations

OS: overall survival
FBW7: F-box and WD repeat domain-containing 7
DCF: docetaxel + cisplatin + 5-FU
SCF: Skp1-Cul1-F-box
PFS: progression free survival
MDR: multiple drug resistance
TSC: tumor stem-like cells

## References

[1] Ueda T, Volinia S, Okumura H, Shimizu M, Taccioli C, Rossi S, Alder H, Liu CG, Oue N, Yasui W, Yoshida K, Sasaki H, Nomura S, Seto Y, Kaminishi M, Calin GA (2010). Relation between microRNA expression and progression and prognosis of gastric cancer: a microRNA expression analysis. The Lancet Oncology.

[2] Jemal A, Siegel R, Xu J, Ward E (2010). Cancer statistics, 2010. CA Cancer J Clin.

[3] Cunningham D, Allum WH, Stenning SP, Thompson JN, Van de Velde CJ, Nicolson M, Scarffe JH, Lofts FJ, Falk SJ, Iveson TJ, Smith DB, Langley RE, Verma M, Weeden S, Chua YJ, Participants MT (2006). Perioperative chemotherapy versus surgery alone for resectable gastroesophageal cancer. The New England journal of medicine.

[4] Luebeck EG, Curtius K, Jeon J, Hazelton WD (2013). Impact of tumor progression on cancer incidence curves. Cancer research.

[5] Yoon C, Park do J, Schmidt B, Thomas NJ, Lee HJ, Kim TS, Janjigian YY, Cohen DJ, Yoon SS (2014). CD44 expression denotes a subpopulation of gastric cancer cells in which Hedgehog signaling promotes chemotherapy resistance. Clinical cancer research.

[6] Shah MA, Janjigian YY, Stoller R, Shibata S, Kemeny M, Krishnamurthi S, Su YB, Ocean A, Capanu M, Mehrotra B, Ritch P, Henderson C, Kelsen DP (2015). Randomized Multicenter Phase II Study of Modified Docetaxel, Cisplatin, and Fluorouracil (DCF) Versus DCF Plus Growth Factor Support in Patients With Metastatic Gastric Adenocarcinoma: A Study of the US Gastric Cancer Consortium. Journal of clinical oncology.

[7] Cunningham D, Starling N, Rao S, Iveson T, Nicolson M, Coxon F, Middleton G, Daniel F, Oates J, Norman AR, Upper Gastrointestinal Clinical Studies Group of the National Cancer Research Institute of the United K (2008). Capecitabine and oxaliplatin for advanced esophagogastric cancer. The New England journal of medicine.

[8] Song S, Ajani JA (2013). The role of microRNAs in cancers of the upper gastrointestinal tract. Nature reviews Gastroenterology & hepatology.

[9] Lin S, Gregory RI (2015). MicroRNA biogenesis pathways in cancer. Nature reviews Cancer.

[10] Liu X, Ge X, Zhang Z, Zhang X, Chang J, Wu Z, Tang W, Gan L, Sun M, Li J (2015). MicroRNA-940 promotes tumor cell invasion and metastasis by downregulating ZNF24 in gastric cancer. Oncotarget.

[11] Hsu KW, Wang AM, Ping YH, Huang KH, Huang TT, Lee HC, Lo SS, Chi CW, Yeh TS (2014). Downregulation of tumor suppressor MBP-1 by microRNA-363 in gastric carcinogenesis. Carcinogenesis.

[12] Li BS, Zuo QF, Zhao YL, Xiao B, Zhuang Y, Mao XH, Wu C, Yang SM, Zeng H, Zou QM, Guo G (2015). MicroRNA-25 promotes gastric cancer migration, invasion and proliferation by directly targeting transducer of ERBB2, 1 and correlates with poor survival. Oncogene.

[13] Zuo QF, Cao LY, Yu T, Gong L, Wang LN, Zhao YL, Xiao B, Zou QM (2015). MicroRNA-22 inhibits tumor growth and metastasis in gastric cancer by directly targeting MMP14 and Snail. Cell death & disease.

[14] Ma F, Song H, Guo B, Zhang Y, Zheng Y, Lin C, Wu Y, Guan G, Sha R, Zhou Q, Wang D, Zhou X, Li J, Qiu X (2015). MiR-361-5p inhibits colorectal and gastric cancer growth and metastasis by targeting staphylococcal nuclease domain containing-1. Oncotarget.

[15] Lee KH, Lin FC, Hsu TI, Lin JT, Guo JH, Tsai CH, Lee YC, Lee YC, Chen CL, Hsiao M, Lu PJ (2014). MicroRNA-296-5p (miR-296-5p) functions as a tumor suppressor in prostate cancer by directly targeting Pin1. Biochimica et biophysica acta.

[16] Welcker M, Clurman BE (2008). FBW7 ubiquitin ligase: a tumour suppressor at the crossroads of cell division, growth and differentiation. Nature reviews Cancer.

[17] Knuutila S, Aalto Y, Autio K, Bjorkqvist AM, El-Rifai W, Hemmer S, Huhta T, Kettunen E, Kiuru-Kuhlefelt S, Larramendy ML, Lushnikova T, Monni O, Pere H, Tapper J, Tarkkanen M, Varis A (1999). DNA copy number losses in human neoplasms. The American journal of pathology.

[18] Inuzuka H, Shaik S, Onoyama I, Gao D, Tseng A, Maser RS, Zhai B, Wan L, Gutierrez A, Lau AW, Xiao Y, Christie AL, Aster J, Settleman J, Gygi SP, Kung AL (2011). SCF(FBW7) regulates cellular apoptosis by targeting MCL1 for ubiquitylation and destruction. Nature.

[19] Yokobori T, Mimori K, Iwatsuki M, Ishii H, Onoyama I, Fukagawa T, Kuwano H, Nakayama KI, Mori M (2009). p53-Altered FBXW7 expression determines poor prognosis in gastric cancer cases. Cancer research.

[20] Ibusuki M, Yamamoto Y, Shinriki S, Ando Y, Iwase H (2011). Reduced expression of ubiquitin ligase FBXW7 mRNA is associated with poor prognosis in breast cancer patients. Cancer science.

[21] Iwatsuki M, Mimori K, Ishii H, Yokobori T, Takatsuno Y, Sato T, Toh H, Onoyama I, Nakayama KI, Baba H, Mori M (2010). Loss of FBXW7, a cell cycle regulating gene, in colorectal cancer: clinical significance. International journal of cancer.

[22] Kurashige J, Watanabe M, Iwatsuki M, Kinoshita K, Saito S, Hiyoshi Y, Kamohara H, Baba Y, Mimori K, Baba H (2012). Overexpression of microRNA-223 regulates the ubiquitin ligase FBXW7 in oesophageal squamous cell carcinoma. British journal of cancer.

[23] Wang L, Ye X, Liu Y, Wei W, Wang Z (2014). Aberrant regulation of FBW7 in cancer. Oncotarget.

[24] Bockhorn J, Dalton R, Nwachukwu C, Huang S, Prat A, Yee K, Chang YF, Huo D, Wen Y, Swanson KE, Qiu T, Lu J, Park SY, Dolan ME, Perou CM, Olopade OI (2013). MicroRNA-30c inhibits human breast tumour chemotherapy resistance by regulating TWF1 and IL-11. Nature communications.

[25] Chen Y, Jacamo R, Konopleva M, Garzon R, Croce C, Andreeff M (2013). CXCR4 downregulation of let-7a drives chemoresistance in acute myeloid leukemia. The Journal of clinical investigation.

[26] Vecchione A, Belletti B, Lovat F, Volinia S, Chiappetta G, Giglio S, Sonego M, Cirombella R, Onesti EC, Pellegrini P, Califano D, Pignata S, Losito S, Canzonieri V, Sorio R, Alder H (2013). A microRNA signature defines chemoresistance in ovarian cancer through modulation of angiogenesis. Proceedings of the National Academy of Sciences of the United States of America.

[27] Garofalo M, Croce CM (2013). MicroRNAs as therapeutic targets in chemoresistance. Drug resistance updates.

[28] Wang Z, Fukushima H, Gao D, Inuzuka H, Wan L, Lau AW, Liu P, Wei W (2011). The two faces of FBW7 in cancer drug resistance. BioEssays.

[29] Ke AW, Shi GM, Zhou J, Wu FZ, Ding ZB, Hu MY, Xu Y, Song ZJ, Wang ZJ, Wu JC, Bai DS, Li JC, Liu KD, Fan J (2009). Role of overexpression of CD151 and/or c-Met in predicting prognosis of hepatocellular carcinoma. Hepatology.

[30] Sheng L, He P, Yang X, Zhou M, Feng Q (2015). miR-612 negatively regulates colorectal cancer growth and metastasis by targeting AKT2. Cell death & disease.

[31] Zhu K, Pan Q, Zhang X, Kong LQ, Fan J, Dai Z, Wang L, Yang XR, Hu J, Wan JL, Zhao YM, Tao ZH, Chai ZT, Zeng HY, Tang ZY, Sun HC (2013). MiR-146a enhances angiogenic activity of endothelial cells in hepatocellular carcinoma by promoting PDGFRA expression. Carcinogenesis.

[32] Shi GM, Ke AW, Zhou J, Wang XY, Xu Y, Ding ZB, Devbhandari RP, Huang XY, Qiu SJ, Shi YH, Dai Z, Yang XR, Yang GH, Fan J (2010). CD151 modulates expression of matrix metalloproteinase 9 and promotes neoangiogenesis and progression of hepatocellular carcinoma. Hepatology.

