# Supplementary material for: miR-363 promotes proliferation and chemo-resistance of human gastric cancer via targeting of FBW7 ubiquitin ligase expression
Source: Oncotarget. 2016 May 4;7(23):35284–92. doi: 10.18632/oncotarget.9169 (PMC5085228; doi:10.18632/oncotarget.9169)
Supplement: Supplementary file 1 [file oncotarget-07-35284-s001.pdf]

miR-363 promotes proliferation and chemo-resistance of human gastric cancer via targeting of FBW7 ubiquitin ligase expression

SUPPLEMENTARY MATERIALS

Statistical analysis

Statistical analysis was performed with SPSS software (18.0; SPSS, Inc., Chicago, IL). Values are expressed as mean±standard deviation (SD). The Student t test was used for comparisons between groups. Categorical data were analyzed by the chi-square or Fisher's exact tests. Correlation analysis was performed among miR-363 and FBW7 mRNA. Cumulative recurrence and survival rates were analyzed using Kaplan-Meier's method and the log-rank test. Cox's proportional hazards regression model was used to analyze independent prognostic factors. P<;0.05 was considered statistically significant.

SUPPLEMENTARY FIGURES AND TABLES

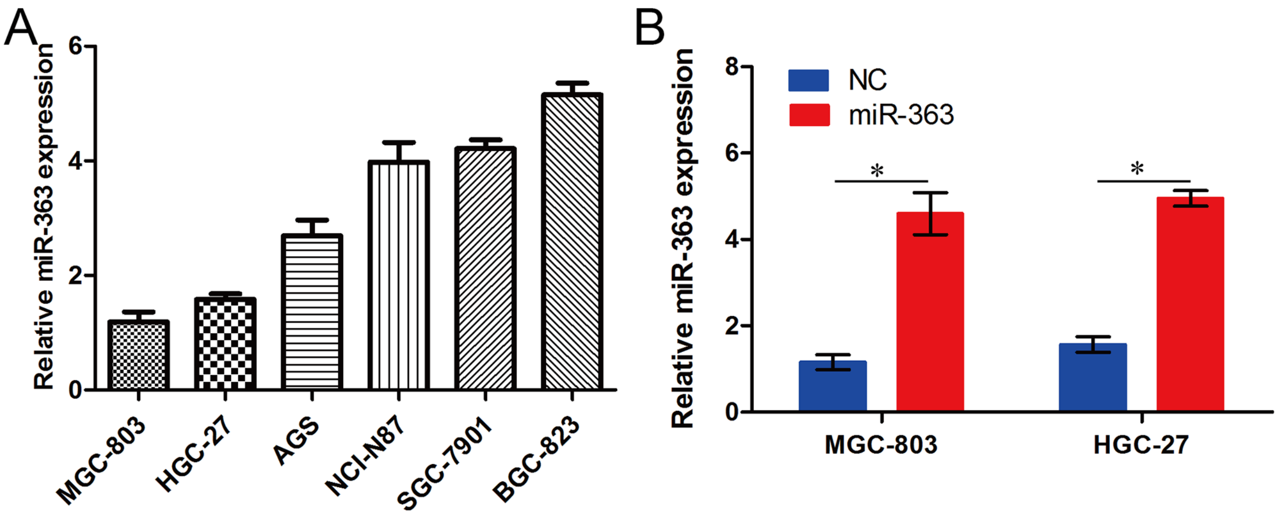

Supplementary Figure S1: Set up forced miR-363 expression gastric cancer cells. A. miR-363 expression in several gastric cancer cell lines was examined using real-time PCR analysis. **B.** Gastric cancer cell lines with forced expression of miR-191 were established.

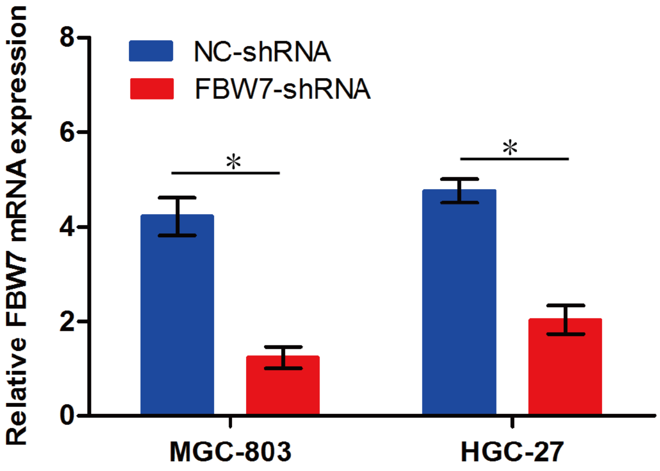

Supplementary Figure S2: Knockdown of the expression of FBW7 in gastric cancer cells using FBW7-shRNA.

Supplementary Table S1: Relationship between miR-363 expression and clinicopathologic parameters of gastric cancer patients

See Supplementary File 1

Supplementary Table S2: Univariate and multivariate analyses of factors associated with survival and recurrence (cohort 1)

See Supplementary File 2

Supplementary Table S3: Univariate and multivariate analyses of factors associated with survival and recurrence (cohort 2)

See Supplementary File 3

Supplementary Table S4: Demographic and baseline characteristics of the patients (DCF-Treated population)

See Supplementary File 4

Supplementary Table S5: The sequence of primers for PCR

See Supplementary File 5
